# Potassium Permanganate in Tuberculous Fistulæ

**Published:** 1910-07-16

**Authors:** 


### Medicine.

### POTASSIUM PERMANGANATE IN TUBERCULOUS FISTULÆ.

There is little or no doubt that oxidation and allied conditions often act inimically upon aërobic as well as upon anaërobic bacterial lesions, and the fact that many a chronic case of empyema is hastened in its healing by oxygen put into the wound by means of a sterilised glass cannula attached to an oxygen cylinder is familiar enough. There are certain other conditions, however, in which the application of oxygen in this way is less easy, and the use of certain oxidising agents may be resorted to instead. The effectiveness of potassium permanganate crystals inserted into a snake-bite immediately after its occurrence is known to many people who have lived in the Tropics. It is probable that potassium permanganate acts in a somewhat similar way when it is used in the manner advocated by Dr. Scobie for the cure of tuberculous fistulæ.

Amongst other cases he had a male patient, twenty-eight years of age, who suffered from tuberculosis of the left lung and from two tuberculous areas in the region of the anus. These were situated to the left of the anal aperture, each being half an inch in length and apparently healed, but examination in a good light showed that they were still discharging a little pus; to the right of the anus there was a bluish unhealthy-looking swelling about the size of a bantam's egg, the residue of an ischio-rectal abscess which had been incised but which had filled up again. When opened it discharged a tablespoonful of thick pus. Careful probing of all three lesions did not reveal anything in connection with the bowel. The patient was in a very emaciated condition, and would allow of no further operative treatment. Crystals of potassium permanganate were introduced into the healthier of the two fistulæ after it had been carefully washed out. The patient experienced a sharp pain lasting about a minute, but there was no other discomfort; the treatment was continued for three weeks, the crystals being allowed to dissolve slowly in the feeble discharge. The result was a perfect healing of the fistula. Dr. Scobie, thus encouraged, packed the other in the same way twice a week, and in two months' time it also healed up finally. The ischio-rectal abscess was now washed out with hot, freshly made permanganate solution, the interior being thereafter filled with crystals of permanganate of potash. The cavity being larger than the others had been, the patient experienced more pain, and this lasted about an hour after the crystals were inserted, but the effect on the discharge was considerable and marked. The general health of the patient continued good, and the cavity steadily shrunk up until presently there was no pus visible except at intervals of a week or ten days, and in due course the wound healed as the others had done.

