# Potassium Permanganate in Tuberculous Fistulæ

**Published:** 1910-07-23

**Authors:** 


A Correction.—We regret that in the article "Potassium Permanganate in Tuberculous Fistulæ," which appeared in our issue of July 16 (p. 462), the source of the information was inadvertently omitted. The original paper, by Dr. Scobie, appeared in *The Prescriber* of April last, to which we are indebted for the abstract.—Ed. The Hospital.

